# Avocado intake and cardiometabolic risk factors in a representative survey of Australians: a secondary analysis of the 2011–2012 national nutrition and physical activity survey

**DOI:** 10.1186/s12937-024-00915-7

**Published:** 2024-01-25

**Authors:** Yasmine Probst, Vivienne Guan, Elizabeth Neale

**Affiliations:** School of Medical, Indigenous and Health Sciences, University of Wollongong, Northfields Avenue, Wollongong, NSW 2522 Australia

**Keywords:** Avocado, National survey, Cardiometabolic risk, Type 2 diabetes mellitus, Australia

## Abstract

**Background:**

Avocados are a rich source of nutrients including monounsaturated fats, dietary fibre and phytochemicals. Higher dietary quality is reported in studies of consumers with higher avocado intakes. The present study aimed to examine avocado consumption and cardiometabolic risk measures in a representative sample of Australian adults.

**Methods:**

A cross-sectional analysis was performed using Australian Health Survey 2011-2013 (*n* = 2,736 observations). Day 1 24-hour recall data was used to examine reported avocado intake (whole avocados and avocado-containing products excluding avocado oil) and cardiometabolic risk measures (LDL, HDL, and total cholesterol, triglycerides, apolipoprotein B, HbA1c, plasma glucose, systolic and diastolic blood pressure). T-tests and chi square analyses were conducted between low (5.21 [95% CI: 4.63, 5.79] grams/day) and high (44.11 [95% CI: 35.89, 52.33] grams/day) consumers of avocado.

**Results:**

14.7% of Australians were ‘avocado consumers’ (n = 403 observations). Mean avocado intake was 24.63 (95% CI: 20.11, 29.15) grams per day, with a median intake of 10.40 (IQR: 4.49–26.00) grams per day for those considered ‘avocado consumers’. Consumers of avocados had a lower BMI and waist circumference (each, *p* ≤ 0.001), lower plasma glucose level (*p* = 0.03), and higher HDL cholesterol (*p* ≤ 0.001) when compared with non-consumers. A trend towards lower plasma glucose, HbA1c (each, *p* = 0.04) and higher dietary fibre intake (*p* = 0.05) was found between high and low consumers of avocado.

**Conclusions:**

Our study suggests favourable outcomes for avocado intake and cardiometabolic characteristics of consumers. Future studies should explore glucose homeostasis using a clinical trial design to understand potential relationships between avocado intake and cardiometabolic risk factors.

## Background

Globally, non-communicable diseases are responsible for 41 million deaths per year with cardiovascular diseases accounting for 23.1% (17.9 million) and diabetes for 3.7% or 1.5 million deaths [1]. The development of cardiovascular disease and diabetes is mediated through complex biological pathways including elevated blood pressure and triglyceride levels, total and low density lipoprotein (LDL) cholesterol and blood glucose biomarkers [2]. The cardiometabolic risk of a person is based on the likelihood of a person developing type 2 diabetes or having a vascular event. Contributing to this, a number of physiological and nutrition related risk factors exist include dyslipidaemia, hypertension, smoking, abdominal obesity, consumption of alcohol above moderate levels, low consumption of fruit and vegetables and ethnicity [3]. A total of 255 million disability-adjusted life-years are attributable to dietary risk factors worldwide [4]. Thus, a better understanding of modifiable risk factors, including diet, are needed.

Studies demonstrate that patterns of eating that follow a whole food approach can have beneficial effects for cardiometabolic health. The Mediterranean pattern of eating is one example that encourages the consumption of a range of whole food items and has demonstrated a decreased incidence and mortality of cardiovascular disease; with meta analyses demonstrating prospective effects attributed to the inclusion of vegetables, legumes and olive oil [5]. Meta-analyses addressing the risk factors for cardiometabolic health have also shown positive effects for waist circumference, high density lipoprotein (HDL) cholesterol and triglyceride levels and no differences for blood pressure or fasting blood glucose levels [6]. Despite the Mediterranean diet being known for the inclusion of fruits, vegetables, whole grain choices, and nuts and seeds, with an emphasis on healthy fats, few studies have report on the inclusion of avocado as a healthy fat component within this pattern of eating [7].

Epidemiological evidence suggests that consumption of avocados aligns to a higher dietary quality compared with those who do not consume avocado [8]. Clinical trial evidence also suggests a positive effect of avocado on total cholesterol levels with overweight adults [9]. A meta-analysis of clinical trial studies reported lower total and LDL cholesterol levels and higher HDL cholesterol levels in the intervention compared with the control groups with no effects on triglyceride or fasting glucose levels [10]. As avocados are recognised as a rich source of key nutrients including monounsaturated fatty acids, dietary fibre, potassium, magnesium, and phytochemicals [11, 12]; and contain approximately 21.6% fat, 1.8% protein and 0.4% carbohydrate [13], they provide a unique nutrient profile within a whole food option.

Contrary to popular belief, lowering intakes of total dietary fat from whole food options or through an overall pattern of eating is not necessary to reduce the risk of cardiometabolic diseases [14]. As avocados are considered to be a medium energy density whole food option, due to the high proportion of water and dietary fibre [12], it is pertinent to explore avocado consumption using an observational study design that can provide added insights towards avocado consumption and risk factors for cardiometabolic disease. Therefore, the aim of this study was to examine avocado consumption and cardiometabolic risk factors in a representative sample of Australian adults. It was hypothesised that consumption of avocados at a population level would be low but would align with reduced cardiometabolic risk profiles of consumers.

## Methods

This analysis extends on our previous examination of avocado intakes in the Australian population and the relationships with food and nutrient intakes [15].

### Study design and participants

The 2011–2013 Australian Health Survey (AHS) is a cross-sectional population-based survey conducted by the Australian Bureau of Statistics (ABS) that sampled urban and rural households across all Australian states and territories. The AHS consists of two separate surveys, the National Health Survey (NHS) and the National Nutrition and Physical Activity Survey (NNPAS) and the National Health Measures Survey (NHMS), a third component for which respondents from both surveys were invited to participate [16]. In the NHS and NNPAS, 21,108 private dwellings (*n* = 18,355 after sample loss during the field stage) and 14,363 private dwellings (*n* = 12,366 after sample loss during the field stage) were selected, respectively. The 2011–2012 NNPAS sampled householders in private dwellings from all Australian states and territories (*n* = 8) applying a multi-staged, stratified-area, probability sampling design. The final sample of the NNPAS included 12,153 Australians aged two years and older. The survey reported a 77% response rate [16]. Anthropometric and blood pressure measures were collected in the NNPAS on a voluntary basis by trained interviewers during home visits. Of the 30,329 respondents aged five years and older in the combined sample (NHS and NNPAS), 11,246 (37.1%) participated in the biomedical component (NHMS). Data relating to the fasted tests relate only to the fasting population (*n* = 7,582).

**Fig. 1:**
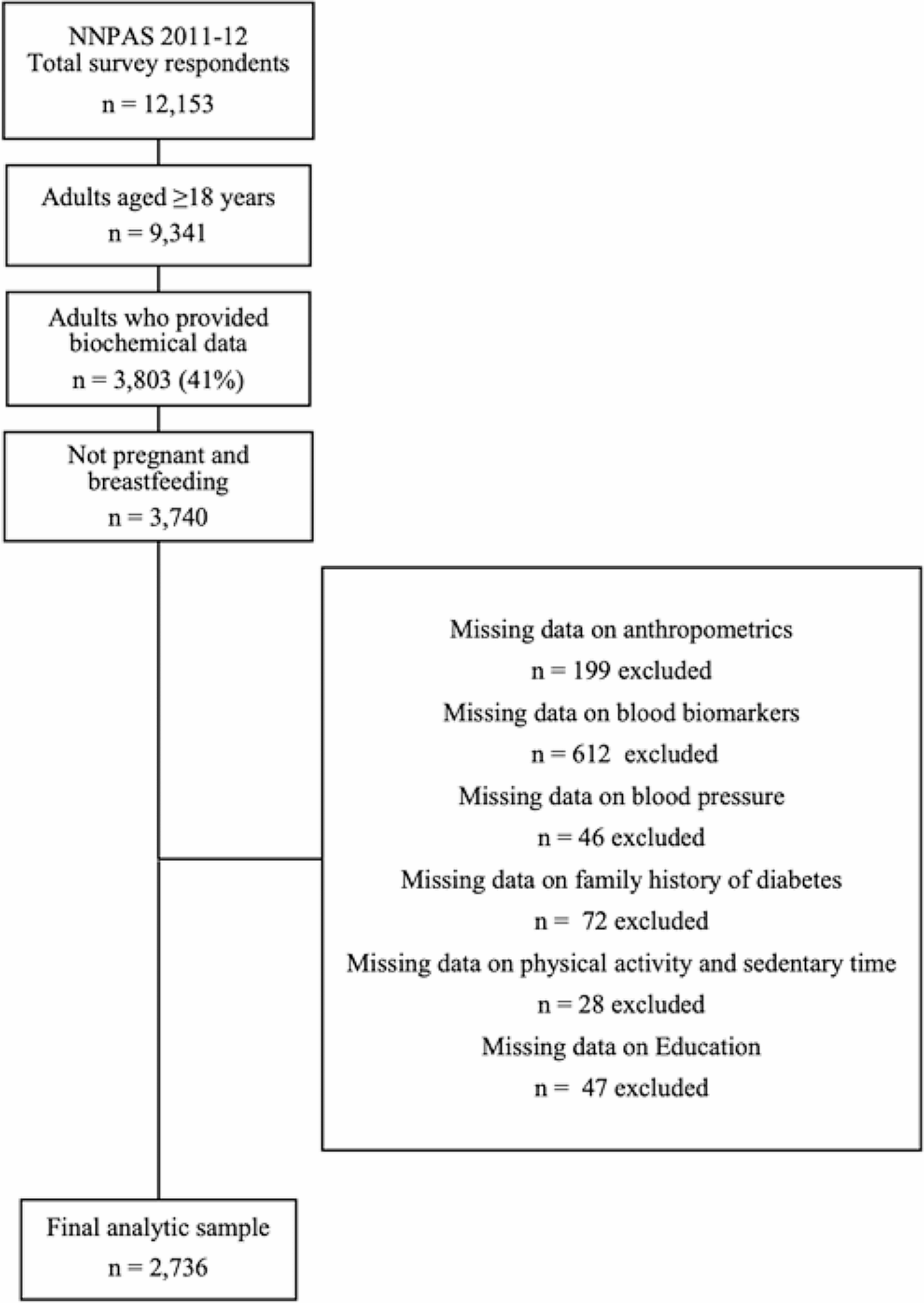
CONSORT diagram of respondents included in the present study

Only data for adult participants, excluding pregnant and breastfeeding females were used in our analyses. Population weights provided by the ABS were applied to ensure that the data was considered representative of the Australian population at the time of the survey. Details of the methods for the survey have been published elsewhere [16] and the data used in our study included a final analytic sample of *n* = 2,736 observations (Fig. 1**).**

### Food and nutrient intake assessment

Dietary intakes data for the NNPAS was collected using two days of 24-hour recall assessment following an automated multiple-pass method (AMPM) [17]. Our study used the data for day one only. Dietary intake data were analysed by Food Standards Australia New Zealand using the Australian nutrient tables (AUSNUT) 2011–2013 food composition database [11]. The key nutrients examined in our study were total fat (grams), saturated fat (grams), monounsaturated fat (grams), polyunsaturated fat (grams), carbohydrates (grams), protein (grams), dietary fibre (grams), vitamin E (mg), magnesium (mg), sodium (mg) and potassium (mg). As no prior national analyses of Australian avocado intake had been reported to be used for comparison, the selection of nutrients for our study was based on those reported by Fulgoni et al. demonstrating variation between ‘avocado consumers’ and ‘non-consumers’ in a representative sample from the United States (US) [8]. Many of these nutrients also fall outside of the recommended intake ranges for Australians [18–22]. Usual intake data for fruit and vegetables were also collected in the NNPAS survey using short questions [16].

### Avocado intake estimation

Avocado (*Persea americana*) is a fruit, shaped similar to a pear, with a green to black shiny outer skin that contains yellow flesh that has a green outer hue [15, 23]. Avocadoes are usually consumed without the skin or the seed. Avocado consumption for our study was estimated using an avocado-specific database that was previously developed by our team [24]. The database was created using the AUSNUT 2011–2013 food composition database [13]. A majority of the nutrient data in the database that is related to avocados were derived from eight samples of Hass avocados that were purchased across five states of Australia [25]. It can, therefore, be assumed that the Hass avocado type is the most related food item to the AUSNUT 2011–2013 listing for whole avocado [13]. The AUSNUT database represents data for food and beverages that had been reported by respondents to the NNPAS [26]. The avocado-specific database, however, attempts to represent all avocado products where avocado is available in its whole food form. Avocado oil is excluded. Therefore, whole avocados and avocado-containing products were included in the analyses of our study reported as grams per day. To calculate the amount of a food item that contained avocado, the percentage avocado of a composite food product was multiplied by the amount (in grams) of the food item reported by each respondent.

### Cardiometabolic risk variables

Total cholesterol (mmol/L), triglycerides (mmol/L) and plasma glucose (mmol/L) data were obtained from blood samples of survey respondents who reported fasting for eight hours or more prior to providing a blood sample [16]. Apolipoprotein B (g/L), HbA1c (mmol/mol) and total and HDL-cholesterol were measured in biological samples without fasting. The Friedewald equation was used to calculate LDL cholesterol from total cholesterol, HDL-cholesterol and fasting triglycerides levels [27]. Individuals with a triglycerides level ≥ 4.5 mmol/L were treated as missing data for the estimation of LDL cholesterol [27]. The reference ranges for total cholesterol were defined as ≥ 5.5 mmol/L, for HDL cholesterol < 1.0 mmol/L for males and < 1.3 mmol/L for females and for LDL cholesterol ≥ 3.5 mmol/L. The ranges for triglycerides were defined as ≥ 2.0 mmol/L and for apolipoprotein B as > 1.3 mmol/L for males and > 1.2 mmol/L for females. Impaired fasting plasma glucose was defined as > 6.0 mmol/L and < 7.0 mmol/L, while HbA1c levels indicative of increased risk of diabetes were defined as 42–47 mmol/mol [16]. Diabetes prevalence was estimated in the AHS by self-reported diabetes diagnosed by a doctor. All analyses in the surveys were conducted by Douglass Hanly Moir pathology (Australia).

Systolic (SBP) and diastolic blood pressure (DBP) measurements were taken on the left arm of respondents. Survey interviewers performed duplicate blood pressure readings using an automated blood pressure monitor. The second reading was used, unless there was a difference greater than 10 mmHg between readings, when a third reading was taken and the second and third readings were averaged. Data for anti-hypertensive and lipid lowering medications were not recorded [16].

### Covariates

Covariates were identified based on the published literature [8, 28, 29]. Information for the respondent sociodemographic characteristics and health behaviours were collected in the NNPAS using interviewer-administered questionnaires [16]. For our study, education level was categorised as low (did not complete high school), medium (completed high school or completed some high school or received a certificate or diploma), or high (having a tertiary qualification). Respondents were categorised by the ABS as meeting or not meeting current Australian physical activity guidelines of 150 min and five sessions per week. Self-reported total minutes spent in sedentary behaviour was defined as time spent sitting or lying down at work, or during transport or leisure activities in the past week. Daily sedentary time (minutes) was calculated as sedentary time divided by seven. We categorised smoking habits as smoker or never smoked and family history of diabetes was dichotomised (yes or no). Central overweight and obesity was measured by the interviewer using a waist circumference (cm) measure taken by placing a metal measuring tape across the top of the umbilicus. Under-reporting of dietary intakes in the NNPAS was evaluated by the ABS using Goldberg cut-off values [16] and the energy intake (EI): basal metabolic rate (BMR) ratio of 1.55. The mean EI:BMR ratio for plausible energy reporters for males and females were 1.50 and 1.49, respectively [16], indicating that more than half of the respondents under-reported their intakes. Under-reporting of intake data are suggested to be influenced by overweight and obesity [16]. To reflect the method employed by ABS, a combination of EI, BMR, self-reported physical activity information (sedentary, low, moderate and high) and body mass index (BMI) were used to represent energy intake misreporting in our study. Weight (kg) and height (cm) measurements were assessed in the NNPAS on a voluntary basis by trained interviewers using digital scales or a stadiometer, respectively. Weight measurements were taken once only. Respondents were encouraged to remove their shoes and any heavy clothing prior to having measurements taken though this was not compulsory, and no correction was applied if they declined. BMI was derived using Quetelet’s metric (kg/m^2^).

### Statistical analysis

Complete case analyses and fasted clinical data were used in our study for clinical relevance. All statistical analyses were conducted using Stata Version 16 (version 16, StataCorp, 2019) under strict ABS data laboratory conditions. To account for the survey design, the sampling process and to provide unbiased estimates, the use of replicate weightings were applied in our analyses using jack-knife resampling [30]. The use of sampling weights in conjunction with consideration of the complex survey design is vital to the analyses, as reported previously [31]. A set of replicate and personal weights were supplied by ABS as part of the Confidentialised Unit Record File (CURF) data. Commands in Stata were run using the complex design model. Details of these commands and the Stata codes for replicate weight in the NNPAS have been published elsewhere [30].

The mean and 95% confidence interval (CI) for avocado consumption (grams per day) were calculated and respondents were categorised into ‘avocado consumers’ (any intake) or ‘non-consumers’, as guided by the methods of Fulgoni et al [8]. Differences between groups were tested using independent samples t-tests. Relationships with continuous variables were tested with linear regression and relationships with categorical variables were tested with chi-square analyses. Respondents were also divided into low and high intake categories of avocado using the median and differences between groups were tested using independent samples t-tests. A *p*-value < 0.05 was considered significant.

## Results

Within our sample (*n* = 2,736 observations), the mean reported avocado intake was 3.95 (95% CI: 3.08, 4.83) grams per day. A total of 14.7% of respondents were considered to be ‘avocado consumers’ and 85.3% were ‘non-consumers’. When our analyses were limited to ‘avocado consumers’ (n = 403 observations), the mean avocado intake was found to be 24.63 (95% CI: 20.11, 29.15) grams per day, with a median intake of 10.40 (IQR: 4.49–26.00) grams per day. For ‘avocado consumers’, the mean reported avocado intake for the low and high intake categories were 5.21 (95% CI: 4.63, 5.79) grams per day and 44.11 (95% CI: 35.89, 52.33) grams per day, respectively. (Table 1)

**Table 1:** Demographic and cardiometabolic characteristics of Australian adults of avocado intake from the Australian Health Survey 2011–2013 survey

Characteristics	All (*n* = 2,736)*	Non consumers (*n* = 2,333)**	‘Avocado consumers’ (*n* = 403)***	*P*-value^a^	Low consumers (*n* = 204)¶	High consumers (*n* = 199)¶¶	*P*-value^b^
**Demographic**							
Age, y	49.07 (48.22, 49.92)	49.12 (48.16, 50.08)	48.80 (46.71, 50.89)	0.79	49.09 (45.31, 52.88)	48.50 (45.21, 51.79)	0.84
Female %	49.88	47.05	66.26	≤ 0.001	52.84	69.02	0.01
Highest level of education, %				0.02			0.52
Low	35.63	35.56	36.05		35.87	30.24	
Medium	36.56	36.96	34.22		28.56	34.39	
High	27.81	25.93	38.63		34.49	36.49	
Smoking				0.11			0.64
Smoker	47.86	48.00	47.12		44.27	42.14	
Never smoker	52.14	50.46	61.85		54.66	58.97	
Physical activity level				0.05			0.81
Sedentary	36.04	37.11	29.87		27.48	27.34	
Low	28.82	27.84	34.28		29.53	33.84	
Moderate	21.36	20.61	25.73		22.58	24.73	
High	13.78	12.91	18.81		19.32	15.19	
Meeting physical activity recommendations %	47.43	45.54	58.39	0.03	56.04	51.11	0.37
Sedentary time, min/d	337.04 (327.31, 346.75)	338.53 (328.33, 348.73)	329.20 (304.96, 353.45)	0.47	343.03 (307.64, 378.41)	315.34 (288.61, 342.08)	0.18
Basal metabolic rate, MJ	6.70 (6.64, 6.75)	6.75 (6.69, 6.81)	6.42(6.25, 6.59)	≤ 0.001	6.51 (6.31, 6.72)	6.33 (6.08, 6.58)	0.23
**Cardiometabolic**							
Family history of diabetes (yes/no), %	29.43	29.39	29.66	0.34	25.33	29.16	0.60
Weight, kg	77.99 (77.20, 78.79)	78.70 (77.81, 79.59)	74.3 (72.01, 76.58)	≤ 0.001	75.19 (72.56, 77.83)	73.40 (69.74, 77.06)	0.43
Height, cm	168.75 (168.30, 169.20)	168.97 (168.44, 169.49)	167.61 (166.29, 168.93)	0.07	168.26 (166.50, 170.02)	166.96 (165.31, 168.61)	0.23
Body mass index, kg/m^2^	27.30 (27.06, 27.54)	27.49 (27.21, 27.77)	26.29 (25.65, 26.93)	≤ 0.001	26.45 (25.55, 27.35)	26.12 (25.13, 27.12)	0.64
Waist circumference, cm	92.78 (92.06, 93.49)	93.34 (92.51, 94.17)	89.82 (87.98, 91.67)	≤ 0.001	90.83 (88.52, 93.14)	88.82 (85.66, 91.97)	0.33
Total cholesterol, mmol/L	5.10 (5.05, 5.14)	5.08 (5.03, 5.13)	5.17 (5.03, 5.30)	0.26	5.18 (5.01, 5.34)	5.16 (4.95, 5.36)	0.89
HDL cholesterol, mmol/L	1.35 (1.33, 1.37)	1.33 (1.31, 1.35)	1.44 (1.40, 1.48)	≤ 0.001	1.48 (1.41, 1.54)	1.40 (1.34, 1.47)	0.13
LDL cholesterol, mmol/L	3.15 (3.12, 3.19)	3.15 (3.11, 3.19)	3.16 (3.05, 3.27)	0.95	3.16 (3.02, 3.30)	3.16 (2.98, 3.34)	0.99
Triglycerides, mmol/L	1.28 (1.25, 1.31)	1.29 (1.26, 1.33)	1.23 (1.12,1.34)	0.30	1.17 (1.07, 1.28)	1.29 (1.11, 1.46)	0.25
Apolipoprotein B, g/L	1.00 (0.99, 1.01)	1.00 (0.99, 1.02)	0.99 (0.95, 1.03)	0.56	1.00 (0.95, 1.04)	0.98 (0.92, 1.05)	0.70
Plasma glucose, mmol/L	5.12 (5.08, 5.16)	5.13 (5.09, 5.18)	5.03 (4.94, 5.12)	0.03	5.13 (4.99, 5.27)	4.93 (4.81, 5.04)	0.04
HbA1c, mmol/mol	36.10 (35.86, 36.33)	36.19 (35.91, 36.47)	35.62 (35.02, 36.22)	0.12	36.38 (35.27, 37.48)	34.87 (34.13, 35.62)	0.04
Systolic blood pressure, mmHg	122.80 (121.88, 123.71)	123.08 (122.18, 123.98)	121.31 (119.11, 123.50)	0.11	121.61 (118.63, 124.59)	121.00 (117.41, 124.59)	0.80
Diastolic blood pressure, mmHg	76.53 (75.96, 77.10)	76.59 (76.04, 77.15)	76.19 (74.59, 77.78)	0.61	75.28 (73.43, 77.13)	77.10 (74.56, 79.64)	0.24

Abbreviations: HDL: High density lipoprotein, LDL: Low density lipoprotein, MJ: Megajoule

Data reported as Mean and 95% confidence intervals

Population weighted equivalence *n = 4,655,426, **n = 3,908,305, *** *n* = 747,121, ¶ *n* = 374,109, ¶¶ *n* = 373,012

^a^*P*-value to compare the variables between two groups in avocado consumption (avocado consumers and non-consumers) using chi-square analysis for categorical data and t-test for continuous data

^b^*P*-value to compare the variables between two groups in avocado intakes (high and low) using chi-square analysis for categorical data and t-test for continuous data

Compared to non-consumers, the ‘avocado consumers’, reported significantly higher of monounsaturated fats, polyunsaturated fats, dietary fibre, vitamin E, magnesium, and potassium (all *p* < 0.05). ‘Avocado consumers’ also reported significantly higher intakes of fruit (1.93 serves vs 1.68 serves, *p* = 0.001) and vegetables (2.71 serves vs 2.46 serves, *p* < 0.05) compared with non-consumers. Among ‘avocado consumers’, no significant differences were observed for reported intakes of nutrients between the low and high intake categories of avocado. (Table 2).

**Table 2:** Avocado-related nutrient intakes for Australian adults of the Australian Health Survey 2011–2013 survey

	All (*n* = 2,736)*	Non consumers (*n* = 2333)**	‘Avocado consumers’ (*n* = 403)***								*P*-value^a^			Low consumers (*n* = 204)¶	High consumers (*n* = 199)¶¶	*P*-value^b^
Total energy, MJ	8.79 (8.58, 9.00)	8.80 (8.58, 9.03)	8.73 (8.23, 9.22)								0.77			8.83 (8.23, 9.43)	8.62 (8.01, 9.24)	0.56
**Nutrients** ^**c**^																
Total fat, g	77.36 (74.88, 79.85)	76.67 (73.93, 79.40)	80.99 (75.46, 86.51)								0.17			80.87 (74.38, 87.37)	81.10 (73.58, 88.62)	0.96
Saturated fat, g	28.90 (27.93, 29.87)	28.98 (27.95, 30.02)	28.48 (26.21, 30.75)								0.68			29.09 (26.40, 31.78)	27.87 (24.82, 30.92)	0.49
Monounsaturated fat, g	29.55 (28.52, 30.58)	29.08 (27.94, 30.23)	32.01 (29.67, 34.34)								0.03			31.48 (28.60, 34.36)	32.54 (29.36, 35.71)	0.58
Polyunsaturated fat, g	12.19 (11.64, 12.74)	11.93 (11.31, 12.54)	13.56 (12.27, 14.86)								0.03			13.32 (11.19, 15.45)	13.81 (12.14, 15.49)	0.72
Carbohydrate, g	232.58 (226.73, 238.43)	234.93 (228.73, 241.14)	220.27 (204.25, 236.29)								0.09			224.78 (205.21, 244.34)	215.75 (194.83, 236.66)	0.46
Protein, g	93.06 (90.80, 95.33)	93.35 (90.78, 95.92)	91.57 (86.11, 97.03)								0.57			92.72 (85.30, 100.14)	90.42 (83.70, 97.14)	0.61
Dietary fibre, g	24.43 (23.73, 25.12)	24.07 (23.40, 24.73)	26.32 (24.32, 28.31)								0.03			24.70 (22.06, 27.34)	27.94 (25.39, 30.50)	0.05
Alcohol, g	14.60 (12.85, 16.34)	14.42 (12.56, 16.28)	15.54 (11.83, 19.26)								0.57			16.25 (10.90, 21.60)	14.83 (9.96, 19.71)	0.69
Vitamin E, g	11.27 (10.84, 11.70)	11.03 (10.58, 11.48)	12.50 (11.44, 13.55)								0.01			12.61 (11.02, 14.21)	12.38 (11.07, 13.69)	0.82
Calcium, mg	847.93 (824.95, 870.91)	838.74 (812.16, 865.32)	895.97 (825.01, 966.93)								0.16			884.09 (796.20, 971.98)	907.88 (806.02, 1009.75)	0.71
Magnesium, mg	353.46 (344.45, 362.47)	348.66 (339.66, 357.66)	378.58 (354.79, 402.38)								0.02			376.13 (341.75, 410.52)	381.04 (351.84, 410.23)	0.82
Sodium, mg	2443.94 (2366.55, 2521.32)	2463.09 (2374.59, 2551.59)	2343.77 (2178.65, 2508.88)								0.22			2300.07 (2043.13, 557.02)	2387.59 (2184.53, 2590.65)	0.59
Potassium, mg	3054.69 (2985.00, 3124.39)	3018.45 (2951.88, 3085.03)	3244.27 (3051.71, 3436.84)								0.02			3151.38 (2900.49, 3402.26)	3337.44 (3112.02, 562.86)	0.19
**Food groups** ^**d**^																
Vegetable, serves	2.50 (2.42, 2.57)	2.46 (2.38, 2.53)	2.71 (2.51, 2.91)								0.02			2.54 (2.31, 2.76)	2.88 (2.57, 3.19)	0.06
Fruit, serves	1.72 (1.67, 1.78)	1.68 (1.62, 1.74)	1.93 (1.79, 2.06)								≤ 0.001			1.89 (1.69, 2.08)	1.96 (1.80, 2.13)	0.53

Abbreviations- obs: observations

Data reported as Mean and 95% confidence intervals

Population weighted equivalence *n = 4,655,426, **n = 3,908,305, *** *n* = 747,121, ¶ *n* = 374,109, ¶¶ *n* = 373,012

^a^*P*-value to compare the variables between two groups in avocado consumption (avocado consumers and non-consumers) using chi-square analysis for categorical data and t-test for continuous data

^b^*P*-value to compare the variables between two groups in avocado intakes (high and low) using chi-square analysis for categorical data and t-test for continuous data

^c^ Assessed using 24-hour recall dietary methodology

^d^ Assessed using short questions

## Discussion

The aim of this study was to investigate avocado consumption and cardiometabolic risk factors in a representative sample of Australian adults. Our study found variable avocado consumption across the Australian population. ‘Avocado consumers’ were more likely to be female, non-smokers, meet physical activity recommendations, have a lower BMI and waist circumference, and have higher HDL cholesterol than non-avocado consumers. ‘Avocado consumers’ were also found to report significantly higher intakes of monounsaturated fats, polyunsaturated fats, dietary fibre, vitamin E, magnesium, and potassium, as well as fruits and vegetables, than non-consumers. While our hypothesis was in part proven regarding low intake of avocado, the size difference for the sample of non-avocado consumers limited the analyses for risk factors for cardiometabolic health.

Our study found a small proportion of the population were reportedly consuming avocados at the time of the NNPAS (14.7%), which, when compared with a representative survey in the US [8], was higher than the reported 3% avocado consumers. The amount of avocado reported amongst ‘avocado consumers’ (24.6 g/day) was, however, lower than that the US sample (70.1 g/day) though when considering the variability of reported intakes (SD: 5.4 g US, 91.2 g AU) it appears that the way avocado is consumed may influence this variation. As both studies used 24-hour recall data, this finding suggests that a different form of dietary assessment such as a diet history is better suited to capturing variability and enable consideration of how avocado is placed in relation to habitual intakes. Despite the small avocado intake amounts reported in our sample, our study demonstrated favourable findings amongst those who reported consuming avocados and also had blood glucose data collected as part of the NHMS.

Studies suggest that elevated blood glucose levels may contribute towards the development of type 2 diabetes mellitus [32]. High blood glucose levels promote an acute inflammatory response and contribute to endothelial damage associated with increased risk of cardiovascular events [33]. Foods deliver nutrients and bioactive compounds that can have varying influences on cardiometabolic disease mechanisms. Food-based management of blood glucose dysregulation, by potentially including avocado within patterns of eating, may be a cost-effective strategy for cardiometabolic disease risk reduction [33].

The findings of our study are consistent with the clinical evidence for avocado consumption and blood glucose regulation [34–38]. For example, replacing the carbohydrate components of a person’s eating pattern with avocado during a breakfast meal, a study showed significantly reduced postprandial glucose levels [38]. However, avocados are largely comprised of fat and protein and limited carbohydrate [13] and blood glucose levels are more immediately influenced by the consumption of carbohydrate. However, dietary fibre, as a component of avocados, may play a role in the blood glucose regulation findings seen in our study. Although avocados contain approximately 7.5% fibre [13], the estimated mean fibre intakes reported in our study were higher in the high avocado intake group when compared with the low avocado intake group. Higher intake of dietary fibre has a positive impact on postprandial glucose control [33]. As noted earlier, previous studies also suggested that avocado consumption is associated with favourable diet quality [8]. Dietary components including micronutrients and phytochemicals can have significant and clinically relevant effects on blood glucose modulation [33]. The combination of these dietary components require more research to support the blood glucose regulation outcomes seen in our study.

Dietary strategies which improve glycaemic control are important for maintaining cardiometabolic health. HbA1c is a biomarker of glucose homeostasis representing average glycaemic control over the past 2–3 months [39], also representing both pre- and post-prandial blood glucose concentrations [39]. Prolonged high blood glucose levels have been related to increased risk of cardiometabolic disease [39]. Given insulin is the primary regulator of carbohydrate, fat and protein metabolism, it plays a pivotal role in glycaemic control [40]. Insulin resistance is a key feature of cardiometabolic disease and a major risk factor on the development of prediabetes and type 2 diabetes mellitus [41]. Additionally, insulin sensitivity is affected by the quality of dietary fats [42]. As avocados are rich in monounsaturated fatty acids they may be a valuable replacement to saturated fatty acids in the diet, potentially helping to improve insulin sensitivity in healthy populations [43]. Incorporating avocado in the diet may, therefore, be a useful option in dietary patterns supporting glycaemic control.

In comparison to the glucose findings, we only found a significant difference for HDL cholesterol between the avocado consumers and non-consumers. No differences were observed for other lipid measures and blood pressure, despite clinical trials reporting improved lipid levels following avocado consumption [12]. While the results of the present cross-sectional analysis cannot be compared directly to clinical trials, the substantial variation in the amount of avocado consumed should be noted. Clinical trials tend to incorporate higher doses of avocado into study diets than that those observed in the current study, even amongst the ‘high’ avocado consumers. As a result, the amounts consumed by the Australian population may not have reached the levels required to demonstrate lower cardiometabolic risk measures.

Compared to non-consumers, ‘avocado consumers’ were found to have higher intakes of fruits and vegetables. These findings are similar to previous analyses of the US population which found improved diet quality was associated with avocado consumption [8]. Future analyses should consider analysis of diet quality in relation to avocado intake using Australian data. Improved nutritional intakes in avocado consumers may be the result of nutrients provided by the avocado, though may also be through avocado being consumed at the same time as other core foods, as shown for other foods such as nuts [44]. These findings provide insights into the context in which avocado is consumed in population diets.

The use of a nationally representative estimates make it possible to generalise findings to the wider population. However, there are several limitations to the present study. Firstly, the present study was a cross-sectional analysis which lacks temporality. Causal relationships between avocado consumption and cardiometabolic risk measures cannot be established and it is possible that the results are due to reverse causation. Secondly, given the proportion of ‘avocado consumers’ was small, the power of analyses is limited. The sample was imbalanced between the ‘avocado consumers’ and non-consumers limiting exploration of relationships within the data and suggesting that the findings should be interpreted with caution. While the dietary intake data used in our analyses was for a single day of intake and collected using the validated AMPM, self-report dietary intake data is prone to recall bias. Avocados are not a habitually consumed food item and, therefore, may not be captured in one day of 24-hour recall. Due to the sample size and the analytical conditions for the analyses imposed by the ABS, the use of habitual intake models as used in our previous AHS analyses [15, 45] were not possible. It should also be noted that this study did not exclude respondents who were misreporting their intakes to maximise the sample. To ensure that avocado intakes could be compared across the population, avocado consumption was presented in grams rather than as a percentage of energy with no energy adjustments. The survey data used in this study did not include information in relation to anti-hypertensive, lipid lowering or glucose lowering medications which may impact the biomedical data use in the analyses. Therefore, the findings of the present analysis should be interpreted accordingly.

## Conclusion

While limited by a small sample, this study has demonstrated favourable cardiometabolic characteristics of avocado compared with non-avocado consumers. Our findings should be interpreted with caution due to the low consumption levels of avocado even amongst avocado consumers. The relationship between avocado intakes and blood lipid profiles warrants further investigation using more advanced statistical models and study designs to better understand potential relationships between avocado intake and cardiometabolic risk factors.

## Data Availability

The data that support the findings of this study are available from the Australian Bureau of Statistics but restrictions apply to the availability of these data, which were used under license for the current study, and so are not publicly available. The Avocado specific database is available at the cited reference provided.

## Abbreviations

ABS: Australian Bureau of Statistics
AHS: Australian Health Survey
AMPM: Automated Multiple-Pass Method
AUSNUT: Australian Nutrient tables
BMI: Body mass index
BMR: Basal metabolic rate
CI: Confidence interval
CURF: Confidentialised Unit Record Files
DBP: Diastolic blood pressure
EI: Energy intake
HbA1c: Haemoglobin A1c
HDL: High density lipoprotein
IQR: Interquartile range
LDL: Low density lipoprotein
NHMS: National Health Measures Survey
NHS: National Health Survey
NNPAS: National Nutrition and Physical Activity Survey
SBP: Systolic
US: United States
